# Research advancements in nanoparticles and cell-based drug delivery systems for the targeted killing of cancer cells

**DOI:** 10.32604/or.2024.056955

**Published:** 2024-12-20

**Authors:** MERYEM A. ABDESSALEM, SIRIN A. ADHAM

**Affiliations:** Department of Biology, College of Science, Sultan Qaboos University, Muscat, 123, Oman

**Keywords:** Drug delivery, Cancer, Nanoparticles, Liposomes, Micelles, Combination therapies, Targeted therapy, Precision medicine, Tumor microenvironment (TME)

## Abstract

Nanotechnology in cancer therapy has significantly advanced treatment precision, effectiveness, and safety, improving patient outcomes and personalized care. Engineered smart nanoparticles and cell-based therapies are designed to target tumor cells, precisely sensing the tumor microenvironment (TME) and sparing normal cells. These nanoparticles enhance drug accumulation in tumors by solubilizing insoluble compounds or preventing their degradation, and they can also overcome therapy resistance and deliver multiple drugs simultaneously. Despite these benefits, challenges remain in patient-specific responses and regulatory approvals for cell-based or nanoparticle therapies. Cell-based drug delivery systems (DDSs) that primarily utilize the immune-recognition principle between ligands and receptors have shown promise in selectively targeting and destroying cancer cells. This review aims to provide a comprehensive overview of various nanoparticle and cell-based drug delivery system types used in cancer research. It covers approved and experimental nanoparticle therapies, including liposomes, micelles, protein-based and polymeric nanoparticles, as well as cell-based DDSs like macrophages, T-lymphocytes, dendritic cells, viruses, bacterial ghosts, minicells, SimCells, and outer membrane vesicles (OMVs). The review also explains the role of TME and its impact on developing smart DDSs in combination therapies and integrating nanoparticles with cell-based systems for targeting cancer cells. By detailing DDSs at different stages of development, from laboratory research to clinical trials and approved treatments, this review provides the latest insights and a collection of valuable citations of the innovative strategies that can be improved for the precise treatment of cancer.

## Introduction

According to Globocan’s 2022 cancer statistics, there were over 20 million new cases of cancer worldwide, with 9.7 million deaths attributed to the disease [1]. Despite the advancement in cancer therapies, there are still many challenges to achieving a complete response in patients [2]. The difficulty in cancer treatment is rooted in the heterogenic nature of the tumors that contain different types of rapidly growing cells with genetic and epigenetic instability and mutations [3,4], the surrounding TME, and their ability to evade the immune system [5,6]. Besides the side effects and toxicity due to cancer treatments, certain chemotherapies can also enhance metastasis [1]. Mechanisms of drug resistance vary from tumor cells’ ability to efflux the drugs [2] to alter their metabolism enhanced by the surrounding hypoxic TME [3–5]. Other mechanisms include cell cycle regulation that targets drugs against specific cell cycle phases [6], bypassing pathways to evade the effects of targeted therapies [7], and DNA repair mechanisms that aid the cancer cells in repairing changes in DNA caused by the drug [8]. Adding to the list of treatment challenges is the development of stubborn cancer stem cells that can alter the drug metabolism and reduce efficacy with added hurdles to all cellular responses [9]. Besides the cellular mechanisms of resistance, patient variability increases the complexity of genetic differences, overall health, and treatment responses, which can affect outcomes, making personalized approaches essential yet challenging to apply [10]. Several treatment approaches were taken to overcome the challenges mentioned above by designing new drugs based on docking to their molecular targets in the cells, giving the term “targeted therapies” to increase specificity and avoid harm to normal cells seen using chemotherapy [7,11,12]. Whether it is chemotherapy or targeted therapy, the same challenge with drug-resistant mechanisms can be seen [13]. Therefore, cancer research in the past two decades focused on developing biotechnological tools based on drug delivery systems (DDS) to overcome the mechanisms of drug resistance essential for administering therapeutic compounds to achieve desired outcomes [14–16]. Conventional drug delivery methods, which are non-targeted, often suffer from significant limitations, including harmful side effects on healthy tissues, low bioavailability, and rapid metabolization of active ingredients upon entering the body [17].

In contrast, smart, targeted DDSs offer a more refined approach by delivering therapeutic agents directly to the site of interest while sparing healthy cells. The precision of drug delivery reduces the required dosage, minimizes side effects, and ensures more uniform drug concentrations in the bloodstream, maintaining therapeutic levels over an extended period [17]. As a result, evolving DDS technology is critical to improving outcomes, particularly for cancer patients. Nanotechnology offered the use of nanoparticles as carriers of single or multiple drugs, and different types of functionalized nanoparticles were proven to increase drug bioavailability, biocompatibility, and pharmacokinetics [18–20]. The success achieved using nanoparticles in improving patients’ survival [21,22] paved the way to develop smart nanocarriers that not only deliver the drug and stabilize its bioavailability but allow the release of their payload in response to specific signals from the TME sensing the TME PH, Hypoxia, levels of enzymes, and adenosine triphosphate, reducing the off-target effects on adjacent cells of the neighboring tissue [23,24]. Smart nanocarriers and many other cell-based delivery systems are developed, and others are in the pipelines to be produced and used to deliver drugs precisely and effectively. An excellent example of a cell-based delivery system is the immune cells equipped and designed to recognize and migrate to sites of infection or inflammation, including the organization of TME through the release of cytokines [25,26]. Since immune cells are naturally part of the immune system, they can be superior in their biocompatibility compared to synthetic carriers, besides their versatility in carrying proteins, small molecules, and gene therapies [27]. Immune cells are used in delivering immunotherapies, or vaccines can be bolstered to fight tumor cells more effectively by enhancing the immune response [28]. Like the smart nanocarriers, engineering immune cells allow for incorporating stimuli-responsive mechanisms, enabling them to release their payload in response to specific signals from the TME [29].

Besides immune cells, nanoscale engineering led to the production of viral-like particles that are investigated for their ability to deliver drugs to cancer cells efficiently [30]. The oncolytic viruses also show promising outcomes when combined with immunotherapy [31]. Not only viruses but also bacteria are engineered with cell-specific antigens for specific recognition and the destruction of tumor cells [32]. Therefore, this review summarizes the latest research output for the available DDSs for the precise targeting of cancer cells and provides the readers easy access to a recent collection of articles that cover DDSs.

## The Role of Nanoparticles and Their Mode of Action in Drug Delivery

Nanoparticles in drug delivery began with the discovery of liposomes in the 1960s [33]. Soon after, dendrimers were invented in 1978 [34], followed by PEGylated liposomes [35] and the development of the first Food and Drug Administration (FDA) approved nano-drug called Doxil™, in 1995 [36,37].

Nanoparticles make for excellent drug delivery platforms due to their improved pharmacokinetic properties [38], controlled rate of drug delivery [39], and their ability to deliver drugs in response to certain stimuli [40]. Due to their small size, nanoparticles and other macromolecules can infiltrate the TME via increased leaky vasculature surrounding the tumor [41,42]. Such particles tend to accumulate more in tumor tissue than in normal tissue [41–43], resulting in an effect termed the enhanced permeability and retention (EPR) effect [44], which has been the basis behind the use of nanodrugs as cancer therapeutic agents [44–46]. Various cancer DDSs based their drug-targeting ability on this passive phenomenon, as it has been argued that the EPR effect is sufficient for targeting drugs to the TME. It has also been argued that it is better than active epitope-targeting as it is more generalized and can be applied to a wide range of tumors [47]. However, some studies question the very basis of the EPR effect. In one study, a 10-year analysis revealed that a median of only 0.7% of injected nanoparticles reached tumor cells via the EPR effect [48]. In contrast, Sindhwani et al. found that number to be even less, as their study showed that tumor accumulation of nanoparticles was 0.63% [49]. The small percentage of nanoparticle accumulation in the tumor can be due to the complex microenvironment around the tumor cells, poor blood flow, and their detection by the immune cells and rapid clearance from the bloodstream [50,51]. There are several strategies to improve the physical and chemical properties of nanoparticles for better penetration into the TME and activate them using different ligands that better target them and make them accumulate in tumor cells [52–54].

It was also suggested that active trans-endothelial mechanisms are the primary method of nanoparticles entering tumors [49]. The cells that facilitate nanoparticle transport into tumors are called nanoparticle transport endothelial cells [55]. That contradicts the initial belief that nanoparticles are passively extravasated via inter-endothelial gaps [56]. The EPR effect was further challenged by Nguyen et al., whose study proposed that nanoparticle retention in tumor tissues is not due to damaged lymph nodes, as lymphatic vessels are the primary nanoparticle exiting the tumor [57]. Instead, they proposed that nanoparticle retention was a consequence of the delay in transport from the nanoparticles’ entry to its exit [57]. That is why it is crucial to target therapeutic nanoparticles to the TME or cancer cells actively. Nanoparticles used for drug delivery exist in various forms, each with their properties, advantages, and disadvantages. Examples include liposomes, micelles, protein-based and polymeric nanoparticles [30,58–60]. Complex drug delivery methods also exist, combining nanoparticles with cell-based systems such as immune cells, viruses, bacteria, and their derivatives [61–63].

## Types of Nanoparticles as DDSs

### Liposomes

Bangham and Horne described Liposomes in 1964 as one of the first nanoparticles used and most studied as DDSs [64]. Liposomes ranging in size from 50–500 nm [65] can be cationic, anionic, or neutral [66]. These spherical vesicles comprise a lipid bilayer encapsulating an aqueous interior core and are produced when amphiphilic lipids are added to water [67]. They are excellent DDSs because they can stably encapsulate hydrophobic, hydrophilic, and amphiphilic drugs. Furthermore, liposomes are nontoxic, hypoallergenic, non-antigenic, and biodegradable [68].

The first liposomes altered to target specific cells had anti-H-2k antibodies conjugated to them. These liposomes displayed specificity in binding to mouse H-2k cells and did not bind to H-2d cells *in vitro* [69].

**Figure 1:**
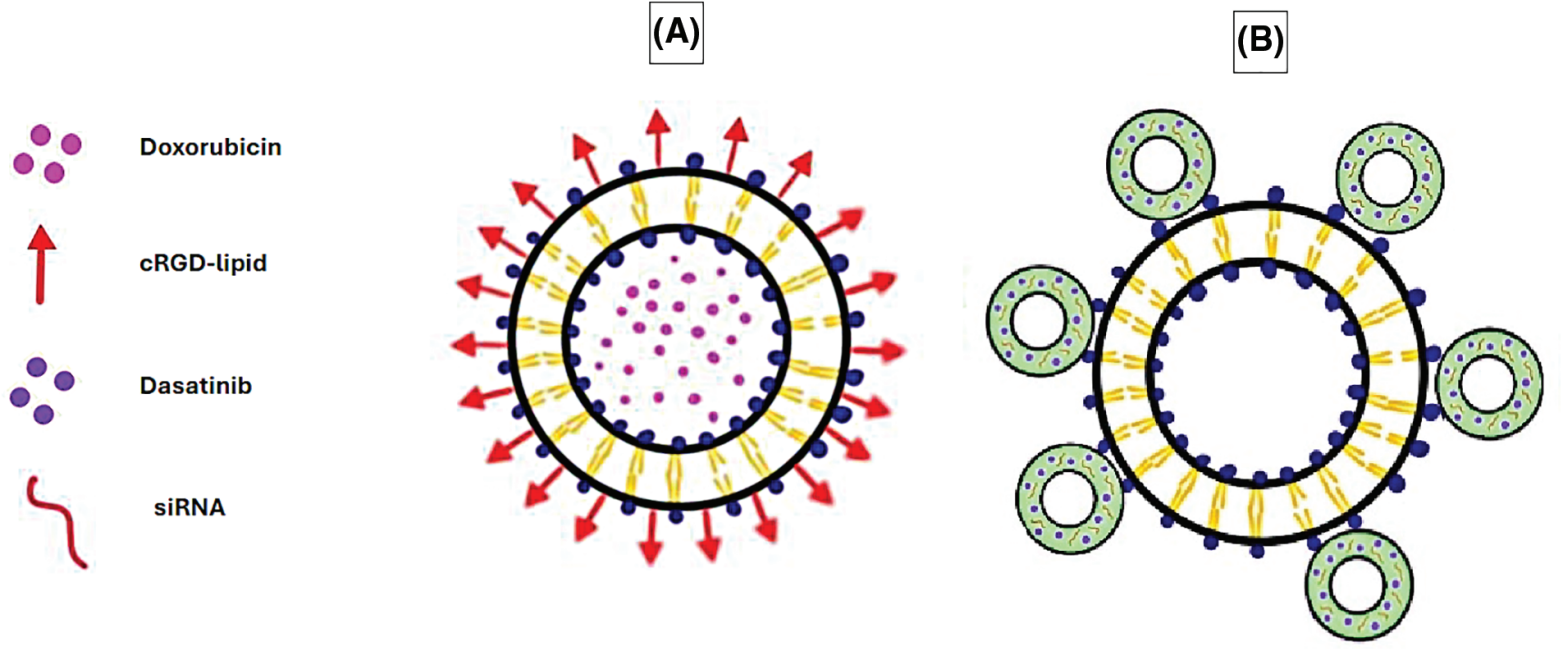
Examples of liposomes used as DDSs in cancer therapy, liposomes *vs*. microbubble liposomes. (A) Doxorubicin-loaded liposome conjugated with cRGD-lipid. (B) Microbubble-liposome loaded with dasatinib and siRNA. The graphing software HiPaint, by Aige (Wuhan) Technology Co., Ltd. (China), was used to prepare the above figure.

Recent studies have successfully fabricated liposomes conjugated to cyclic arginine-glycine-aspartate (cRGD), which are pH-sensitive and loaded with doxorubicin (Fig. 1A). The cRGD liposomes have been tested both *in vitro* on the A-549 lung carcinoma cell line and *in vivo* on nude mice bearing A-549 tumors and have exhibited excellent antitumor activity by inhibiting cell proliferation. The liposomes also displayed a high selectivity towards A-549 cells and low toxicity towards regular HL-7702 cell lines in both *in vitro* and *in vivo* studies. These modified liposomes also displayed a rapid cellular uptake, sustained intracellular drug release, greater retention at the tumor site, and notable pH sensitivity as they degraded in the acidic TME, releasing doxorubicin [70].

The cRGD liposomes were taken one step further in an orthotopic triple-negative breast cancer (TNBC) mouse model with a 4T1 cell line *in vivo*, where the liposomes were co-loaded with paclitaxel, a chemotherapeutic drug, and celecoxib, a selective cyclooxygenase-2 inhibitor. The results demonstrated an enhanced tumor targeting efficiency and accumulation, along with dendritic cell (DC)-mediated immunity, which led to immunogenic cell death at the tumor site and the inhibition of tumor rechallenge and metastasis [71].

Additionally, microbubbles (gas bubbles) have been combined with liposomes to form enhanced DDSs. Microbubbles are pressure-sensitive drug carriers that release their contents upon exposure to acoustic radiation, such as ultrasound. Combined with liposomes, they result in efficient drug delivery where the microbubble delivers the drug intracellularly, whereas the liposome enables high drug doses to be loaded [72]. This system has been utilized to co-deliver the multi-kinase inhibitor dasatinib, along with COL11A1-targeting small interfering ribonucleic acid (RNA) (siRNA) to the lung adenocarcinoma A-549 cell line (Fig. 1B). This study’s outcome exhibited how the dasatinib-siRNA microbubble-liposomes significantly inhibited cell proliferation *in vitro* compared to dasatinib alone or dasatinib microbubble-liposomes lacking siRNA [73].

### Micelles

Micelles are amphiphilic molecules composed of a hydrophilic outer capsule and a hydrophobic core, enabling hydrophobic drugs to be encapsulated within the core [74]. They are formed when lipids or surfactants are dispersed in an aqueous solution, forming aggregates with a size range of 5–100 nm [60,75]. The term micelle was first introduced by James William McBain at a Faraday meeting in 1913 while describing aggregates of soap molecules in aqueous solutions [76,77].

In one study, Fan et al. examined the ability of polyzwitterionic micelles loaded with paclitaxel to inhibit tumor growth after oral administration. The study was conducted *in vitro*, where the micelles successfully penetrated and accumulated in 4T1 tumor spheroids. Furthermore, three *in vivo* mouse models were used, consisting of hepatocellular carcinoma HEP G2 cell line, patient-derived hepatocellular carcinoma xenografts, and TNBC 4T1 cell line, where apoptosis-related death was displayed, and tumors were inhibited with low toxicity to the body [78].

**Figure 2:**
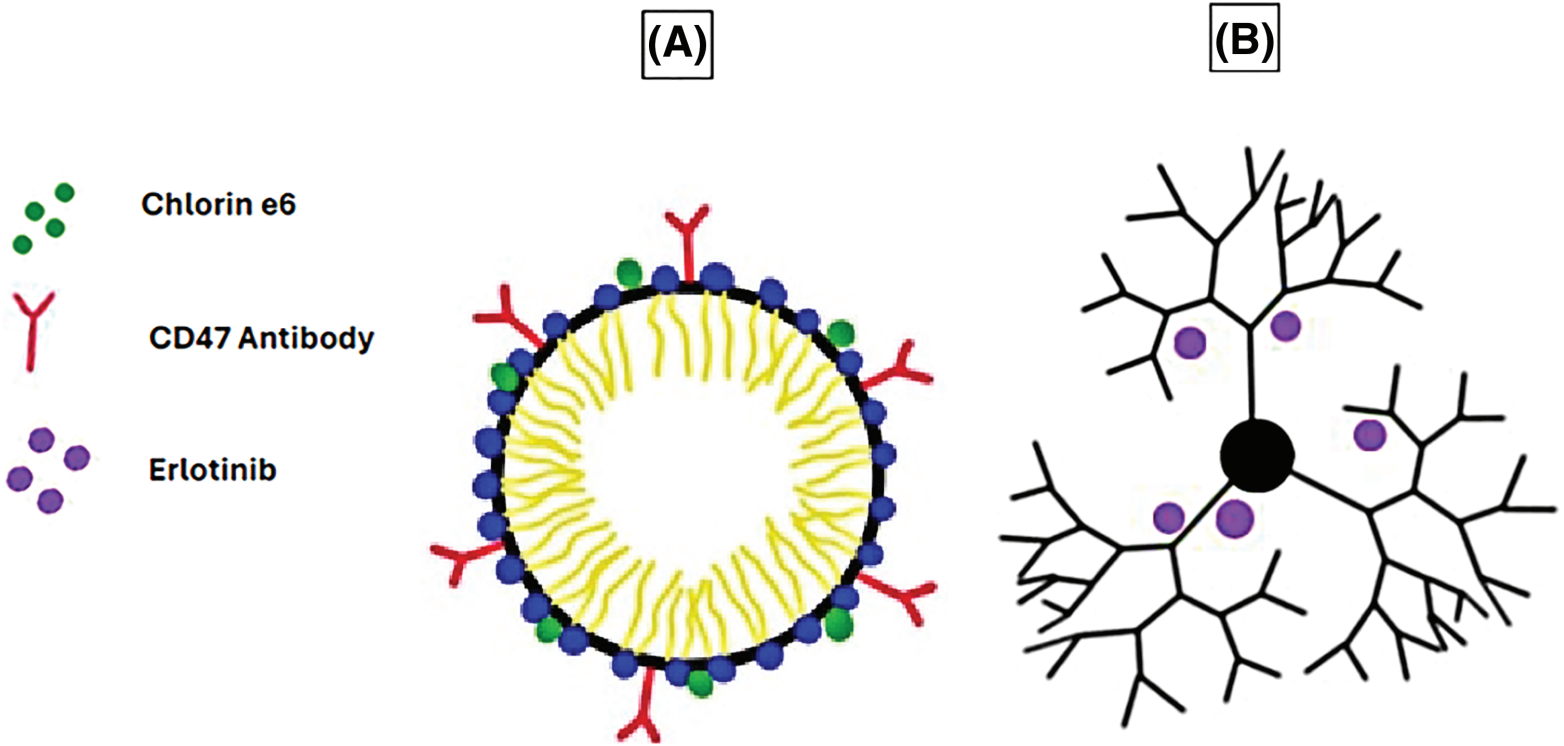
Examples of micelles and dendrimers used as DDSs in cancer therapy. (A) Micelle conjugated with CD47 antibody and loaded with chlorin e6. (B) Erlotinib-loaded G4 PAMAM dendrimer. The graphing software HiPaint by Aige (Wuhan) Technology Co., Ltd. was used to prepare the above figure.

In another study, pH-sensitive micelles co-loaded with CD47 antibodies and chlorin e6 (Ce6) were used to target osteosarcomas in the K7M2 cell line *in vitro* and *in vivo* (Fig. 2A). The acidic TME triggered the release of the micelles’ cargo, leading to the blockage of the “don’t eat me” signals by cancer cells and inducing damage by the reactive oxygen species generated by Ce6, resulting in apoptosis, necrosis, and immunogenic cell death [79].

### Protein-based nanoparticles

Protein-based nanoparticles involve a protein as a major component of their structure [80]. The synthesis methods of protein-based nanoparticles are discussed by Wang et al. [81]. A successful example used for cancer treatment is Abraxane, which combines the chemotherapeutic drug paclitaxel with human serum albumin (HSA), an approved drug to treat breast cancer by the FDA [82]. Pegaspargase (Oncaspar^®^), a PEGylated formulation of asparaginase, is an FDA protein-based nanoparticle that is approved for the treatment of acute lymphocytic leukemia in children [83] with low toxicity and enhanced benefit [84]. Gelatin nanoparticles are another example of a protein-based nanoparticle that research evidence is giving promising results in the delivery of the 3-alkyl pyridinium salt, which inhibits the anti-apoptotic and proliferative effects of nicotine on A-549 lung adenocarcinoma cell lines [85]. Silk fibroin, a biopolymer extracted from domestic silkworms approved by the FDA as a safe and nontoxic product, is employed to deliver bioactive compounds [86]. Experiments also provide evidence for the enhanced efficacy of sorafenib when combined with camel-based casein nanoparticles delivered against hepatocarcinoma cells [87].

### Polymeric nanoparticles

Polymeric nanoparticles are solid particles that consist of macromolecular polymers [88]. One example of such nanoparticles includes dendrimers, which Fritz Vogtle discovered in 1978 [34]. These are three-dimensional hyperbranched polymers with a size range of 1–15 nm [89] and spherical geometry [90]. They consist of a central core with repeated branches attached and a functional group at the periphery [91], allowing them to encapsulate hydrophilic and hydrophobic drugs. Dendrimers are recognized for their reproducibility at a scalable level due to their mono-dispersity [90].

In one study, polyamidoamine (PAMAM) dendrimers of generation 4.5 (G4.5) conjugated with histidine and cysteine delivered doxorubicin to the cervical carcinoma HeLa cell line *in vitro*. The results indicated high specificity towards the cancer tissue with reduced off-target side effects, leading to the inhibition of cell proliferation. The dendrimers also inhibited cell proliferation in zebrafish models *in vivo* [92].

Another study compared the use of two separate erlotinib-loaded PAMAM dendrimer generations, G4 (Fig. 2B) and G5, and their ability to function as DDSs. This study, conducted on non-small cell lung carcinoma (NSCLC) A-549 cell line *in vitro*, suggested that the erlotinib-loaded G4 dendrimers had a more significant impact on cell viability than the erlotinib-loaded G5 dendrimers. Moreover, the erlotinib-loaded G4 dendrimers had a significant uptake by the cells compared to the G5 dendrimers, which had minimal cellular uptake [93].

Another example of polymeric nanoparticles is chitosan, which is a linear polymer composed of *N*-acetyl-D-glucosamine and β-(1-4)-linked-D-glucosamine [94] and obtained from the partial deacetylation of the parent polymer, chitin [95]. The synthesis of chitosan nanoparticles from the chitin polysaccharide usually present in exoskeletons of arthropods [96] with various sizes can be done by numerous procedures. Still, the most used methods are ionotropic gelation and polyelectrolyte complex [97]. Functionalizing the chitosan into nanoparticles has shown promising results when used as a DDS against cancer cells [98]. A study has examined the cytotoxicity of black pomegranate-loaded, chitosan-coated magnetic nanoparticles on breast cancer MDA-MB-231 and 4T1 cell lines *in vitro*. The results suggested that these nanoparticles successfully induced more significant cell line toxicity than the free drug. Furthermore, it was revealed that the nanoparticles had no toxic effect on regular NIH/3T3 cell lines [99]. Other synthetic polymeric nanoparticles showed high specificity to cancer cells, such as the synthetic copolymer that included folic acid with poly (styrene-alt-maleic anhydride) (FA-DABA-SMA) via a biodegradable linker 2,4-aminobutyric acid (DABA) to target folic acid receptors on cancer cells [19,20,100]. A list of the different types of nanoparticles can be found in Table 1 below.

**Table 1:** A comparison among the types of nanoparticles as DDSs

Nanoparticle drug delivery system	Description	Size	Charge	Drug-loading
Liposomes	Spherical vesicles that are composed of a lipid bilayer encapsulating an aqueous interior core [67]	50–500 nm [65]	Varied [66]	Hydrophilic and hydrophobic drugs [68]
Micelles	Amphiphilic molecules are composed of a hydrophilic outer capsule and a hydrophobic core [74]	5–100 nm [60]	Neutral [101]	Hydrophobic drugs [102]
Protein-based nanoparticles	Nanoparticles made out of protein	Variable [103,104]	Variable [105]	Hydrophilic and hydrophobic drugs [106]
Polymeric nanoparticles	Nanoparticles made out of various polymers	Variable [97]	Variable [20]	Hydrophilic and hydrophobic drugs [100]

## Cell-Based DDSs

### Immune cells as DDSs

Immune cells are equipped innately with toll-like receptors (TLRs), NOD-like receptors (NLRs), and RIG-I-Like Receptors (RLRs), serving as cell surface proteins that can detect invaders such as bacteria, viruses, or pathogens alerting the immune system to act by releasing the cytokines [107–109], this system is also found to target cancer cells and destroy them however, cancer cells express some of these receptors that can exploit signaling to promote their survival or suppress immune responses [109,110]. Dendritic cells, macrophages, and T lymphocytes, also known as T Cells, infiltrate the TME innately to react with tumor cells [111–113]; this ability of communication between the immune cells and cancer cells allows them to be the best candidates for genetically engineering them with specific chimeric antigen receptors (CARs) that are designed with motifs to recognize the cancer antigens, bind to them and eventually destroy them [114]. Therefore, Encapsulating CAR T cells with liposomes or lentiviruses makes them vehicles to deliver their payloads specifically to the tumor site [115]. In the TME, tumor-associated macrophages (TAMs) exist in one of two polarization forms: the pro-inflammatory M1 form, which targets and engulfs tumor cells [116], or the immunosuppressive M2 form, which promotes cancer progression via the secretion of anti-inflammatory cytokines [117].

#### Macrophages and their derivatives in drug delivery

Adoptive cell therapy is defined as introducing immune-competent cells as a treatment modality for cancer. Building upon macrophages’ natural affinity to inflammation in the TME, they have been studied as delivery agents for cancer therapeutic drugs. In one report by Qiang et al., a DDS was created by loading doxorubicin-loaded reduced graphene oxide (rGO) into a mouse macrophage-like cell line, RAW264.7. This system utilized both the innate tumor-targeting ability of macrophages, along with ‘rGO’s photothermal properties to synergistically target and inhibit the growth of tumor cells both *in vitro* in using RM-1, a mouse prostate cancer cell line and *in vivo* in mice bearing RM-1 tumors, via the rapid heat-induced release of doxorubicin from rGO when exposed to near-infrared (NIR) radiation [118]. A similar study was conducted by Nguyen et al. with macrophages co-loaded with citric acid-coated superparamagnetic nanoparticles and doxorubicin-loaded thermosensitive liposomes. The experiments were performed *in vitro* on the TNBC 4T1 cell line and *in vivo* on a mouse model, concluding that the artificially generated magnetic field allowed the macrophages to travel to the tumor’s location [119].

In addition to directly loading macrophages with therapeutic nanoparticles or drugs, some scientists have reported using macrophage byproducts or components to enhance cancer drug delivery. One example includes the use of exosomes obtained from macrophages. The exosomes were used to co-deliver doxorubicin and cholesterol-modified microRNA (miRNA) 159 *in vitro* to MDA-MB-231 cells and on a TNBC *in vivo* mouse model, resulting in a synergistic therapeutic effect. Antitumor effects were exhibited, such as reduced cell proliferation and invasion, increased apoptosis, the silencing of transcription factor 7 (TCF7), and the protein levels of both TCF-7 and Myc [120]. In another study, Haney et al. used macrophage-derived extracellular vesicles (EVs) to deliver the chemotherapeutic drugs doxorubicin and paclitaxel to MDA-MB-231 TNBC cells *in vitro* and in both T11 and MDA-MB-231 mouse models *in vivo*. The EVs exhibited efficient accumulation and antitumor activity *in vitro* while inhibiting tumor growth *in vivo* [121]. Despite their low immunogenicity, high stability, affinity to tumor cells, and ability to fuse with other cells under acidic TME conditions, there are limitations to these EVs. Such limitations include significant alterations to the EV structure needed for efficient drug loading and the restriction of cargo carried by EVs, as they should be hydrophobic [121].

#### CAR-T and dendritic cell-based vaccines

The success of getting FDA approval for using a CAR-T cell drug named Kymriah to deliver tumor-specific antigens is a milestone in supporting personalized medicine for patients with acute lymphocytic leukemia [122,123]. Infiltrated T-cells can be isolated from tumors and cultured as active tumor targets. Once the T-cells are activated and propagated *ex vivo*, they will be returned and infused into the same patient from whom they were initially isolated; therefore, they are called autologous antitumor lymphocytes [124]. Recent studies suggested that CAR-T cells overcome the chemotherapy resistance in hard-to-treat cancers and increase overall survival [125]. Like engineered T-cells, DC-based Vaccines are another type of DDS, such as immune cell therapy. DCs are genetically modified to express tumor antigens and are usually more effective at triggering antitumor-immune responses when compared to DCs pulsed with tumor peptides [126]. The DC vaccine named PROVENGE is a successful example of an FDA-approved prostate cancer DC vaccine [127].

### Viruses as DDSs

Viruses are obligate intracellular parasites [128] that infect and reproduce inside host cells [129]. They consist of short segments of either deoxyribonucleic acids (DNA) or ribonucleic acids (RNA) [130] encapsulated within a protein layer called a capsid [129]. Viruses can further be divided into two groups: enveloped viruses, bound by an outer lipid membrane, and non-enveloped viruses that ‘don’t have a lipid membrane [131].

Viral nanoparticles (VNPs) comprise materials obtained from plant, bacterial, and mammalian viruses to form the drug-enclosed capsid protein [132]. They also retain the innate qualities of viruses, such as their ability to self-assemble into capsids, while carrying artificial cargo, such as therapeutic drugs [129]. On the other hand, virus-like particles (VLPs) are a sub-class of VNPs [133] that are non-infectious as they lack genetic materials [134]. These viral particles can induce immune responses via their antigenic properties and are thus also used in vaccine development [129].

**Figure 3:**
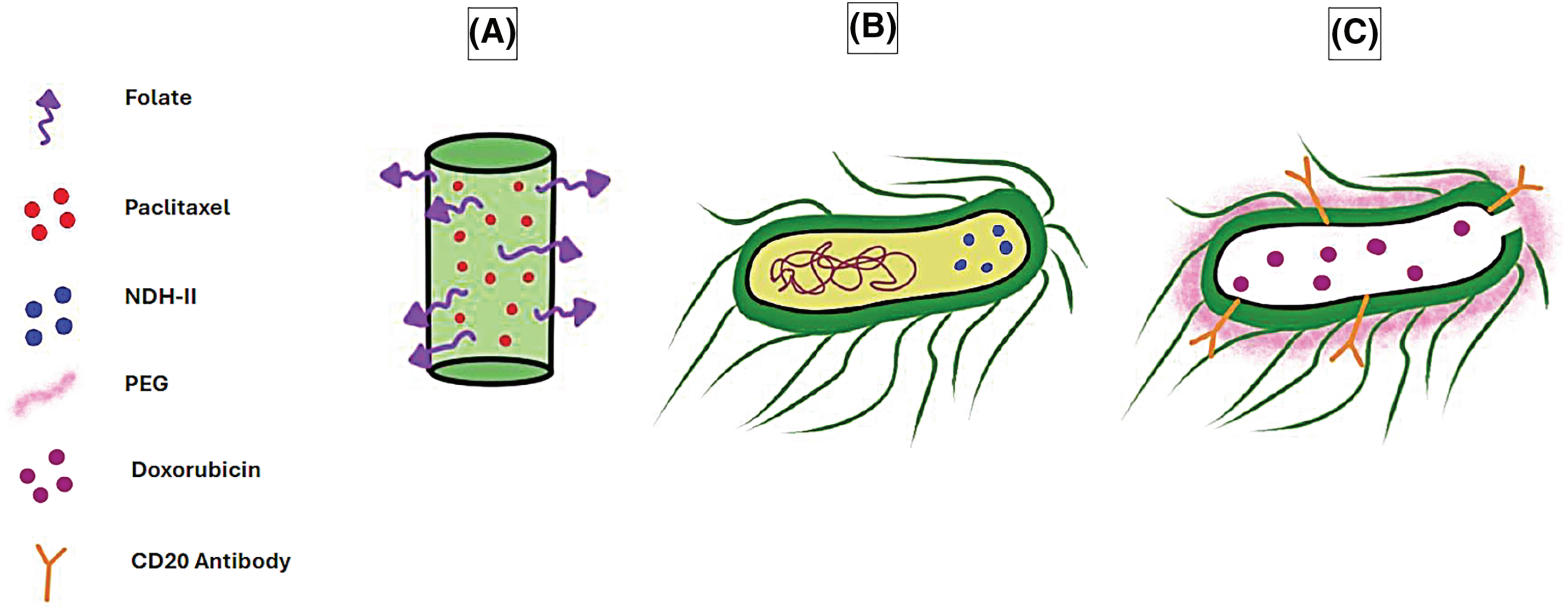
Examples of viruses and bacteria used as DDSs in cancer therapy. (A) Paclitaxel-loaded, folate-conjugated PMMoV. (B) *E. coli* engineered to overexpress NDH-II. (C) Doxorubicin-loaded PEG-coated bacterial ghost conjugated with CD20 antibody. The graphing software HiPaint, by Aige (Wuhan) Technology Co., Ltd., was used to prepare the above figure.

In one study, particles of a plant virus called pH-sensitive pepper mild mottle virus (PMMoV) were conjugated with folate and loaded with paclitaxel to form FA-PMMoVs (Fig. 3A). These viral nanoparticles were then compared with free paclitaxel and targeted toward breast cancer MCF-7 cell line *in vitro*, with the results suggesting enhanced cellular uptake and apoptotic cell death in the folate receptor-overexpressing MCF-7 [135].

Moreover, Bao et al. reported using a VNP called reBiosome, packaged with a therapeutic Cas9 messenger RNA (mRNA) gene editing system to delete an oncogenic enhancer of c-Myc in the MCF-7 breast cancer cell line. The outcome indicates successful gene deletion, thus a reduction in c-Myc expression and significant inhibition of the MCF-7 cell line *in vitro*. Meanwhile, the *in vivo* results demonstrated tumor growth suppression and apoptotic cell death within the tumor tissue in mice [136].

### Bacteria and bacterial-derived methods as DDSs

Bacteria are another example of vehicles that can deliver antitumor drugs.

Utilizing bacteria to fight cancer isn’t new, as it has been practiced and proven efficient when Willian B. Coley injected a patient with *Streptococcus* to treat their inoperable cancer, leading to tumor shrinkage [137]. He has since treated nearly 1000 patients using bacteria-derived products, termed Coley’s Toxins [138]. Coley’s Toxins stimulate the patient’s immune system, thus helping with tumor destruction via immunotherapy [139]. Although the FDA banned Coleys’ Toxins in 1963 [140], the idea of immunotherapy still sparked in the brains of interested scientists. The immunotherapy drug named axalimogene filolisbac (AXAL, or ADXS11–001) was produced by the attenuation of a strain of *Listeria monocytogenes* (*Lm*) that has been engineered to express the *Lm*-LLO-E7 antigen against human papillomavirus (HPV) infected cancer cells, consequently helping the immune system to target tumor cells infected with HPV [141]. AXAL or ADXS11–001 is currently undergoing clinical trials in various cancer types, including cervical (Phase III) [142–146], head and neck (Phase II) [147,148], and anal cancer (Phase II) [149,150].

Bacteria have been genetically modified by harnessing the power of synthetic biology to target tumor cells and deliver therapeutic agents precisely. They are a great choice due to their innate ability to colonize and proliferate within the hypoxic tumor core [151,152].

In one example, *Escherichia coli* (*E. coli*) was engineered to overexpress alternative NADH dehydrogenase (NDH-II) (Fig. 3B), hence enabling the bacteria to function as a bioreactor running a Fenton-like reaction and producing H_2_O_2_ and hydroxyl radicals, which induced apoptosis in tumor cells. The bacteria successfully targeted the colon cancer CT 26 cell line both *in vitro* and in a mouse model *in vivo* while displaying a reduced accumulation in non-target organs [153].

Additionally, the use of bacteria as DDSs was further employed by Asensio-Calavia et al., where synthetic biology was utilized to program bacteria to target epidermal growth factor receptor (EGFR) on cancer cells and inject catalytic fragments of adenosine diphosphate-ribosyltransferase toxins into colon cancer HCT-116 cell line *in vitro* and *in vivo*, leading to cancer cell death and enhanced tumor suppression [154].

#### Bacterial ghosts (BGs)

BGs are an example where altered bacteria are employed as drug delivery vehicles. BGs, as the name suggests, are empty bacterial ‘shells’ formed from the empty envelopes of Gram-negative bacteria such as *E. coli*. These BGs are formed by expressing the cloned gene E of bacteriophage PhiX174 origin in *E. coli*, which leads to bacterial lysis and the subsequent formation of a transmembrane tunnel through the cell, leaving an intact cell envelope consisting of an outer and inner membrane while lacking a cytoplasm [155]. Recombinant BGs can be formed by engineering the bacteria before subjecting them to lysis, such that recombinant proteins are inserted into the inner membrane by N or C terminal anchor sequences [156].

BGs have several advantages when used as drug delivery agents, including their immense safety, as they are essentially membranes with no cytoplasmic or genetic content. Furthermore, they are affordable to produce in large quantities and can be genetically modified to express any antigen of choice. Finally, they are highly immunostimulatory, which can enhance the effectiveness of the engineered antigen [157].

One recent example of BGs used as anti-cancer drug delivery agents has been demonstrated by Li et al., where doxorubicin-loaded *E. coli* BGs have been modified with polyethylene glycol (PEG) and a CD20 antibody (Fig. 3C) and tested against non-Hodgkin’s lymphoma cells *in vitro*. The results demonstrated great specificity of the BGs, as they targeted CD20-positive Raji cells and exhibited higher cytotoxic effects than BGs that were not modified with a CD20 antibody. Additionally, the modified BGs exerted minimal cytotoxicity on mouse fibroblast L929 cells, representing non-cancerous cells [158].

Similarly, *Klebsiella pneumonia* has been used to form BGs co-loaded with doxorubicin and phycocyanin extract, which resulted in a synergistic antitumor effect when used to treat NSCLC cell lines A-549 and NCI-H358 *in vitro* where these BGs induced apoptosis, inhibited proliferation, modulated oncogenic signaling pathways and increased the cells’ sensitivity to doxorubicin [159].

#### Minicells and SimCells

Minicells, discovered by Adler et al., are miniature, anucleate cells that do not divide and range in size from 100–400 nm [46,160]. Originally obtained from *E. coli* K12 strains, these cells contain proteins and RNA and can respire due to enzyme activity. They cannot divide due to the lack of chromosomal DNA [46]; however, they often contain plasmids [161]. Minicells are produced in rod-shaped bacteria due to unusual cell division at chromosome-free polar ends [161,162]. Such cell divisions in bacteria are usually caused by alterations in the Min system which is the division leading to unequal size of daughter cells, leading to cell wall formation near the cells’ poles rather than at the center of the cells, therefore resulting in two different daughter cells: one small minicell lacking in chromosomal DNA and one elongated, filamentous cell containing chromosomal DNA [163,164].

In a recent study, minicells derived from the probiotic bacterial strain *E. coli* Nissle were engineered to produce azurin, a peptide with potent anti-cancer activity [165]. This study conducted various *in vitro* tests with two colon cancer cell lines: mouse CT-26 and human HT29. The minicells exhibited their ability to preferentially target and bind to the tumor cells compared to the colon epithelial NCM-460 cell line, leading to the release of azurin at the TME and apoptotic cancer cell death. Additionally, the azurin-minicells inhibited cancer cell proliferation, migration, and invasion [164]. Bacteria minicells were tested in Phase I clinical trials, where minicells engineered to target EGFR have been tested on patients with advanced solid tumors and patients with recurrent glioblastoma [166,167].

**Figure 4:**
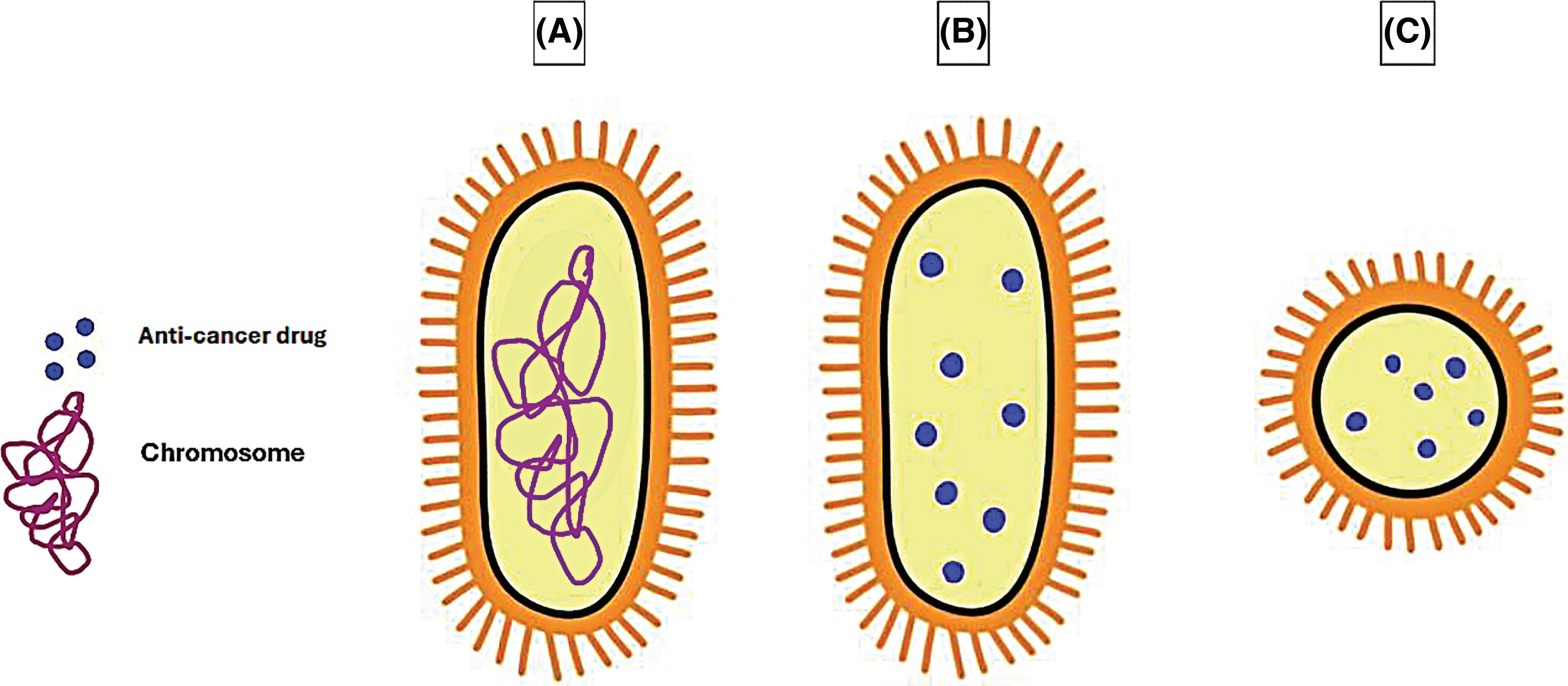
Comparison in the size of a (A) normal bacterial cell, (B) drug-loaded Sim Cell, and (C) drug-loaded minicell. The graphing software HiPaint, by Aige (Wuhan) Technology Co., Ltd., was used to prepare the above figure.

SimCells are generated novel, simple cells, which are cells that build on the idea of minicells that lack a chromosome. The difference between minicells and SimCells is their size (Fig. 4A–C) and the method by which the chromosomes have been removed. SimCells are normal-sized cells genetically engineered to express I-CeuI, an endonuclease that forms double-stranded breaks in the bacteria’s chromosome. After the formation of the double-stranded breaks, the chromosome will be degraded entirely via the bacterial cell’s endogenous nucleases [168].

In one study, SimCells displaying anti-carcinoembryonic antigen (CEA) nanobodies demonstrated their ability to target the CEA-expressing colorectal Caco-2 cell line and not the non-CEA-expressing SW-80 cell line. Additionally, these SimCells were engineered with an inducible genetic circuit to produce the anti-cancer drug catechol, which resulted in cancer cell death *in vitro* and a precise manner [169].

#### Bacterial outer membrane vesicles (OMVs)

OMVs were first observed by Knox et al. as blebs bound by triple-layer membranes released by *E. coli* grown under conditions of limited lysine [170]. OMVs are released from gram-negative bacteria during their exponential growth phase [171]; these spherical vesicles have a size range of 20–500 nm [172,173] and are found in the bacterial growth media [171]. OMVs are obtained from the bacteria’s outer membrane, and their contents are similar to those of the bacteria they come from [174], as they contain outer membrane and periplasmic proteins but at a lower concentration when compared to the parent cells [175]. OMVs also contain RNA and chromosomal DNA [176] while lacking cytoplasmic proteins [175].

Compared to other DDSs, OMVs are advantageous due to their innate immunogenicity, as they include both immunogenic endogenous proteins [177] and lipopolysaccharides (LPS) on their surface [178]. Additionally, they preferentially target and accumulate in tumor tissues over healthy ones and exhibit antitumor effects on various cancer types, as suggested by studies on numerous *in vivo* mouse models with the following cell lines: MC-38 colon cancer, metastatic 4T1, B-16BL6 melanoma, and CT-26 colon adenocarcinoma [179] and *in vitro* against cell lines including breast cancer MCF-7 and colon cancer Caco-2 [180]; breast cancer SkBr3 and leukemia Nalm6 [181]; and ovarian cancer OV-33 [182]. In contrast, standard cell lines such as human keratinocytes HaCaT and human dermal fibroblast HdFn were less affected by the OMV’s cytotoxicity [182,183].

To date, various studies have utilized OMVs as DDSs. In one example, OMVs were engineered to display human epidermal growth factor receptor-2 (HER-2) affibodies, small proteins that selectively bind to targets with a high affinity [184]. These OMVs, loaded with kinesin spindle protein siRNAs, lead to cell death of various HER-2 expressing cell lines *in vitro*, including ovarian cancer SKOV-3, breast cancer BT-474, and HCC-1954. Interestingly, only the siRNA-loaded OMVs caused cytotoxicity and not the unloaded OMVs. The tests were conducted on mouse models *in vivo*, where the affibody siRNA-loaded OMVs displayed more significant tumor inhibition than the non-targeted OMVs [185].

OMVs have been engineered to display various proteins that aid in targeting them towards tumor cells, including programmed death-1 (PD-1) to target tumor cells overexpressing programmed death ligand-1 (PD-L1) in mouse models derived from B-16 melanoma and CT-26 colorectal cell lines. The results were promising as the PD-1 OMVs had more potent tumor inhibition than PD-1 alone or OMVs alone [186].

A similar idea was described in Pan and colleagues’ work, where they developed OMVs displaying LyP-1 to target tumors *in vitro* and deliver PD-1 plasmids. The three cell lines tested (4T1 breast cancer, B-16 melanoma, and CT-26 colorectal cancer) successfully took up the OMVs and expressed PD-1, which bound to their intrinsic PD-L1, thus leading to self-blockade. The potent anti-tumoral activity was seen both *in vitro* and *in vivo* [187].

Another interesting example can be seen in Rezaei Adriani et al.’s study, where OMVs were engineered with scFv proteins to target EGFR on various cell lines *in vitro*, such as HT29 colorectal cancer cells, HCT-116 colon cancer cells, A-549 lung cancer cells, 4T1 breast cancer cells, and normal kidney HEK-293 cells; and in 4T1 mouse models *in vivo*. The *in vitro* results did not display any cytotoxicity. In contrast, the *in vivo* results showed significant antitumor effects explained by the OMVs’ activation of natural killer (NK) cells that skewed M2 macrophages towards an M1 phenotype in mice, and these NK cells are not present in the *in vitro* cell culture model [188].

Finally, one interesting article described macrophage-encapsulated OMVs used to deliver Ce6 and doxorubicin to breast cancer 4T1 cell lines *in vitro* and in a mouse model *in vivo*. The therapeutic outcome consisted of shifting macrophages from anti-inflammatory M2 to pro-inflammatory M1 and pyroptosis, a form of microbial-initiated inflammatory cell death [189,190]. The list of cell-based drug delivery systems is listed in Table 2 below.

**Table 2:** A comparison among the types of cell-based DDSs

Cell-based drug delivery system	Description	Size
Immune cells	Utilizing the patient’s immune system to deliver drugs and fight cancer cells [191]	Up to 50 μm [192]
Viruses	Utilizing virus or virus-derived proteins to encapsulate and deliver drugs and fight cancer [132]	20–200 nm [193]
Bacterial ghosts	Empty bacterial ‘shells’ formed from the empty envelopes of gram-negative bacteria [155]	Length: 1–2 μm
		Width: 0.5–2 μm [194]
Minicells	Miniature, anucleate cells that do not divide due to the lack of chromosomal DNA [46]	100–400 nm [195]
	Produced in rod-shaped bacteria as a result of unusual cell division at chromosome-free polar ends [161,162]	
Simcells	Normal-sized cells that have been genetically engineered to degrade their chromosomes [168]	620 nm–2 μm [196]
Bacterial outer membrane vesicles	Spherical vesicles are defined as blebs bound by triple-layer membranes [170]	20–500 nm [197]
	Released from gram-negative bacteria during their exponential growth phase [171]	

### Nanoparticles combined with cell-based DDS results in better therapeutic effect

Combination therapies employ multiple therapeutic agents to treat cancer [198] and reduce the drug resistance commonly caused by monotherapies [199]. Since drug combinations are effective in cancer treatment, using a good DDS is needed to carry multiple drugs to the TME, which is one of the significant objectives of current research. Many of the laboratory studies discussed in this review utilized the co-delivery of drugs (combination therapy) that showed better results in combating cancer cells, such as combining microbubbles with liposomes [71–73].

Similarly, the co-delivery of paclitaxel and resveratrol combination using liposomes and macrophages showed a better ability to target tumor cells when compared with using liposomes alone in an *in vivo* mouse study that is attributed to the innate homing ability of the macrophages [200]. Drug-loaded nanoparticles maintain macrophages’ viability and homing ability, as the free drugs could be toxic. Moreover, the macrophages should be derived from the same patient to minimize harmful side effects [118]. Interestingly, combining bacterial ghosts with nanoparticles gives promising results, as shown by Ling et al., who examined the use of liposomal paclitaxel-loaded BGs coated with cancer cell membranes. This combination therapy was tested on the 4T1 cell line *in vitro* and on a metastatic lung cancer mouse model *in vivo*, leading to positive results that demonstrated synergistic chemo-immunotherapy. Furthermore, the cancer cell membrane enabled a faster fusion of the BGs with tumor cells compared to the BGs on their own and a higher cancer cell targeting by the nanoparticles [201].

## Discussion

Indeed, recent developments in personalized and precision medicine have been achieved due to the development of smart delivery systems. Research and development are improving the DDSs based on the challenges faced at the laboratory or clinical levels. All indicators show that the future of cancer therapy may lie in combining nanotechnology and cellular engineering, bringing hope for better treatment efficacy with minimum toxicity for such heterogeneous diseases. The diversity of DDSs discussed in this review provides a collective view of the different types and options for researchers to choose from to deliver drugs effectively to tumor cells. Each DDS has its mechanism of action, and despite the diverse DDSs we see, the ones first invented are still in use and show effectiveness, such as the FDA-approved Doxil and Onivyde [202]. Research and development continue to improve liposomes to customize and enhance their interactions with negatively charged cancer cells while reducing their toxicity to healthy cells bearing a neutral charge [203]. The drug-loading capacity of liposomes is also relatively large—more significant than that of micelles and similar to that of dendrimers, allowing a larger quantity of drugs to be loaded if needed [204].

Concerning using micelles as DDSs, there are a few disadvantages, including their comparatively low drug-loading capacity [203], their ability to carry only hydrophobic drugs efficiently [102], their poor stability in the blood [205], and the insufficient interactions between micelles and malignant cells due to their neutral charge [203]. Despite these drawbacks, the main benefit of micelles is their low toxicity [206].

Dendrimers are another type of DDS discussed in this review. Although dendrimers have advantages, such as their high drug-loading capacity [203] and reproducibility at a scalable level [90], they also have a few downsides. Their main issue is their toxicity, especially for dendrimers containing amine groups, such as PAMAM dendrimers. Dendrimers with a higher generation tend to be more toxic than their lower generation counterparts, as amine groups increase as a dendrimer’s generation rises [207]. Another thing to consider is the dendrimer’s charge, which can be altered to carry either a positive, negative, or neutral charge [208]. Notably, positively charged dendrimers function as a double-edged sword because although they induce cytotoxicity in negatively charged cancer cells [209], they are also toxic to normal cells [210] and erythrocytes [211].

Similar to other bacterial-derived DDSs, such as BGs, the surface of OMVs is decorated with LPS. The benefits of the LPS displayed on OMVs are a topic of great controversy among scientists. In one aspect, the lipid A component of LPS is responsible for its endotoxicity and can lead to sepsis and toxic shock [178]. On the other hand, the LPS also leads to beneficial immunostimulatory effects in the TME. Therefore, a balance has to be reached between these two effects [212].

On the bright side, scientists have devised ways to reduce the LPS cytotoxicity in bacteria [213], enabling the OMVs derived from them to be used in cancer therapy [188,189]. The need to reduce LPS toxicity was further demonstrated in a study on the human monocyte THP-1 cell line. This study examined the difference in cytotoxicity between OMVs obtained from msbB *E. coli* mutants *vs*. wild-type strains, and the results indicated that the wild-type strains induced toxicity *in vitro* and even mortality in mice *in vivo* [185].

OMVs are also advantageous due to their innate cytotoxic effects against cancer cells [179,214]. However, a contradicting study suggests that OMVs obtained from pathogenic strains of *E. coli* have no cytotoxic effects on Caco-2 colon carcinoma cell lines and even cause a significant increase in their growth [215]. One issue with OMVs is their contribution to the immune evasion of cancer cells [216], as OMVs induce interferon-γ (IFN-γ) production by T-cells [179], which leads to the overexpression of PD-L1. That, in turn, binds to PD-1 on T-cells, hence suppressing its immune response [186]. Interestingly, two studies indicate that OMVs do not elicit antitumor effects *in vitro* but may do so *in vivo* [186,188]. That might suggest that their *in vivo* toxicity mainly stems from the surrounding immune system’s reaction to OMVs.

Finally, an emerging enhancement to DDSs involves using neoantigens, newly formed antigens from genetic mutations typically found in cancer cells. These mutations produce abnormal proteins not found in normal cells, allowing the immune system to recognize them as foreign. Since neoantigens are unique to the tumor, they are considered promising targets for cancer immunotherapy, as they help the immune system to distinguish between healthy and cancerous cells [217].

Yet, there are hurdles and limitations to the research in targeted drug delivery systems. Factors such as TME barriers represented by the heterogeneity of solid tumors limit the proper delivery of drugs to all areas of the tumor since some areas can be hypoxic, areas with poor blood flow, areas with different interstitial fluid pressure, and regions with varying densities of cells [218–220]. Even though immune cells can target tumor cells, a solid tumor can be immunosuppressive for the immune cells to penetrate and target tumor cells, a limitation scientists are trying to solve using different strategies [220,221]. One difficulty scientists face in targeting nanoparticle use is the circulation clearance by the macrophages in the liver and spleen, which decreases the availability of nanoparticles in the tumor tissue [222,223]. Although PEGylated nanocarriers have shown better efficacy, they face challenges in producing anti-PEGylated antibodies that target these nano-carriers and clear them [224]. Tumor cells adapted to immune cell therapeutics by decreasing the target antigens’ presentation and initiating anti-apoptotic pathways, making it harder for the immune cells to recognize them [5]. In addition to tumor interaction with DDSs, the variability among patients adds another layer of challenges based on the patient’s metabolic activity, immune system function, and tumor characteristics. Therefore, active research looks for all the strategies available based on the DDSs they used to overcome these limitations and challenges. Future meta-analysis for each DDS and the corresponding strategy for improvement is recommended to comprehend all the findings in one place.

## Conclusion

In conclusion, the diverse nature of DDSs indicates a positive sign of hope for better treating the different types of cancer precisely with the most negligible toxicity possible. More efficient cancer treatment can be designed and developed by combining the biocompatibility feature of immune cells and the increase in drug bioavailability of nanoparticles. Strategies to increase the bioavailability and enhance nanoparticle accumulation and action in tumors are under intense research. The active targeting of nanoparticles by ligands, such as peptides, antibodies, or host cell membranes, increases the efficacy of the nanoparticle availability in the tumor tissue. The addition of ligands will add superiority to the nanoparticles by either attaching them to the cells’ membrane or even internalizing them inside the tumor cells. Similar to the ligand binding for active targeting, modification to the physicochemical properties of nanoparticles is critical for the best performance against tumor tissue. Whether it is a nanoparticle, immune cell, virus, or bacteria-derived DDSs, all these vehicles will be designed and chemically or genetically modified to target tumors precisely based on tumor cell surface antigens and their TME. While there are approved drugs using nanoparticles and immune cells for certain types of cancer, the bacterial ghosts, Minicells, SimCells, and bacterial outer membrane vesicles (OMVs) are still under research focus. Since next-generation sequencing technology is accelerating, we believe more DDSs will come along for future trials. Finally, combining nanoparticles with cell-based delivery methods such as immune cells might be a good strategy for the stability and specificity of these delivery vehicles by functioning as a Trojan horse and evading detection by the host’s defense systems.

## Data Availability

No data was used for the research described in the article.

## Abbreviations

ADXS11-001: Axalimogene filolisbac
AXAL: Axalimogene filolisbac
BGs: Bacterial Ghosts
CAR-T: Chimeric Antigen Receptor T-Cell
Ce6: Chlorin e6
CEA: Carcinoembryonic Antigen
cRGD: Cyclic arginine-glycine-aspartate
DC: Dendritic Cell
DDSs: Drug Delivery Systems
DNA: Deoxyribonucleic Acid
EGFR: Epidermal Growth Factor Receptor
EPR: Enhanced Permeability and Retention
EVs: Extracellular Vesicles
FDA: Food and Drug Administration
HER-2: Human Epidermal Growth Factor Receptor 2
HPV: Human Papillomavirus
HAS: Human Serum Albumin
IFN-γ: Interferon-γ
LPS: Lipopolysaccharide
miRNA: Micro RNA
mRNA: Messenger RNA
NDH-II: Alternative NADH Dehydrogenase
NIR: Near Infrared Radiation
NLRs: NOD-Like Receptors
NSCLC: Non-Small Cell Lung Carcinoma
OMVs: Outer Membrane Vesicles
PAMAM: Polyamidoamine
PD-1: Programmed Death 1
PD-L1: Programmed Death Ligand 1
PEG: Polyethylene Glycol
PMMoV: Pepper Mild Mottle Virus
rGO: Reduced Graphene Oxide
RLRs: RIG-I-Like Receptors
RNA: Ribonucleic Acid
siRNA: Small Interfering RNA
TAMs: Tumor-Associated Macrophages
TCF-7: Transcription Factor 7
TLRs: Toll-Like Receptors
TME: Tumor Microenvironment
TNBC: Triple Negative Breast Cancer
VLPs: Viral-Like Particles
VNPs: Viral Nanoparticles
